# Olfaction and thyroid hormones in patients with subjective cognitive decline, non-amnestic and amnestic mild cognitive impairment

**DOI:** 10.1007/s00508-024-02431-4

**Published:** 2024-09-18

**Authors:** Sania Nasserzare, Johann Lehrner

**Affiliations:** Department of Neurology, Medical University of Vienna, Währinger Gürtel 18-20, 1097 Vienna, Austria

**Keywords:** Cognitive dysfunction, Alzheimer’s disease, Olfaction disorder, Smell, Thyroid hormones, Thyrotropin, Thyroxine

## Abstract

**Background:**

Thyroid hormones may affect olfaction in different stages of cognitive impairment: subjective cognitive decline (SCD), non-amnestic (naMCI) and amnestic mild cognitive impairment (aMCI). Additionally, biometric parameters, depression, and neuropsychological performance are considered as possible influencing factors.

**Design and patients:**

A retrospective single-center data analysis was conducted during the observation period 2001–2023, with *n* = 495 (52.3% female) SCD, naMCI and aMCI subjects, aged ≥50 years, at the General Hospital of Vienna.

**Measurements:**

The criterion olfactory function was objectively measured by Sniffin’ Sticks© odor identification and subjectively through the Assessment of Self-Reported Olfactory Functioning test. Serum thyroid hormone levels, mainly thyroid-stimulating hormone, as well as T3, T4, fT3, and fT4, were used to assess thyroid function. Statistical analyses using IBM SPSS® 29.0.0 covered adjusted multiple linear regression models with hierarchical blocks to predict olfactory performance considering β‑weights.

**Results:**

Of the study participants, 4.2% had hypothyroidism and 2.4% had hyperthyroidism. The majority exhibited normal thyroid function. One third (33.5%; 95% confidence interval, CI 29.4–37.0%) were hyposmic. The results indicate no substantial association between thyroid and olfactory functions. Increasing age (β = 0.20), lower performance in the Neuropsychological Test Battery Vienna (NTBV) dimensions verbal memory (β = −0.33) and attention (β = −0.12) appear to be risk factors for lower olfaction. A discrepancy between subjective and objective olfaction was found.

**Conclusion:**

Thyroid and olfactory functions had no substantial relationship. Higher fT4 correlated weakly with lower odor identification. Increasing age and decreased performance in two out of six NTBV dimensions are relevant prognostic factors for olfactory dysfunction.

## Introduction

Dementia is emerging as one of the most serious problems in care of old people. As the mean age of the population increases, the incidence and prevalence of this condition continue to steadily rise worldwide [1]. Alzheimer’s disease (AD) is the most common form of dementia, accounting for 60–80% of cases [2]. Endocrine abnormalities, such as insulin resistance, elevated cortisol, and low estrogen and testosterone levels, are increasingly implicated in the pathogenesis of AD. The most acknowledged connection between endocrine and cognitive functions revolves around thyroid hormones [3]. Serum thyroid-stimulating hormone (TSH) is a standard parameter recommended for assessing thyroid function in dementia screening guidelines [4].

The current evidence links thyroid dysfunction to an increased risk of AD and other forms of dementia [5]. Studies mostly associate cognitive impairment with hypothyroidism, reporting its connection to faster cognitive decline [6, 7]. Conversely, some research suggests that hypothyroidism may be associated with a lower risk of cognitive impairment [8]. Although less documented, hyperthyroidism is also linked to dementia. Studies suggest that subclinical hyperthyroidism may be associated with a higher risk of dementia than subclinical hypothyroidism [9]. Increased dementia risk is associated with TSH levels below the normal range and higher fT4 levels [10].

However, some studies reported no relation between TSH and AD [11]. Van Vliet et al. (2021) found that neither subclinical hypothyroidism nor hyperthyroidism was associated with cognitive function, cognitive decline, or incident dementia, thus questioning the current guidelines of thyroid function screening in the context of cognitive decline [12]. The conflicting results illustrate the current disagreement between aspects regarding the association between thyroid hormones and cognitive decline, particularly in AD.

Olfactory dysfunction (OD) often precedes classical AD symptoms by several years and affects 85–90% of patients in the early stages of the disease [13]. An OD can result from various factors, including thyroid dysfunction, most notably hypothyroidism [13]. Reduced olfactory function (OF) has been reported in adults with primary hypothyroidism, with serum fT3 levels being the most important factor [14]. Furthermore, studies have shown that the treatment of hypothyroidism improves the sense of smell and taste [15]. Olfactory dysfunction is not only found in AD but also with high prevalence in Parkinson’s disease, Lewy Body Disease (LBD) and other dementias [36].

As thyroid hormone changes may be a possible risk factor for AD and OD is one of the earliest symptoms of AD, finding a correlation between the two may provide two inexpensive early biomarkers.

The primary objective was to determine whether thyroid hormone abnormalities have an explanatory value for reduced OF at different stages of early cognitive impairment, particularly in subjective cognitive decline (SCD) and non-amnestic (naMCI) and amnestic mild cognitive impairment (aMCI) patients.

It was hypothesized that abnormal TSH levels can explain reduced OF. Secondary hypotheses were that other parameters, such as biometric and neuropsychological characteristics, also provide an explanatory value.

## Material and methods

### Design

This study presents a university-based, single-center retrospective data analysis. Data collected from cognitively impaired patients of the Department of Neurology of the Medical University of Vienna from January 2001 to January 2023 were examined. Participant-related data were obtained from the Research Documentation and Analysis (RDA) system of the Medical University of Vienna and the general hospital information management (Allgemeines Krankenhaus Information’s Management, AKIM) platform. The data of a total of *N* = 741 patients were available. The study protocol adhered to the Declaration of Helsinki and received approval from the Medical University of Vienna Ethics Committee.

### Data management and participants

Data were gathered from patients who consulted the Memory Outpatient Clinic regarding memory concerns. The patients were assigned either by a physician or through self-referrals.

The participants underwent physical, neurological and neuropsychological examinations, standard laboratory testing (including serum thyroid parameters), and neuroimaging to rule out cerebrovascular diseases. Neuropsychological tests included the mini-mental state examination (MMSE), Vienna visuo-constructional test (VVT.03), vocabulary test (Wortschatz Test, WST-IQ), Beck Depression Inventory (BDI-II) and the Neuropsychological Test Battery Vienna (NTBV). Sociodemographic characteristics, such as age, sex, and education in formal school years, were noted. The OF was measured using Sniffin’ Sticks© of Burghart Instruments and the Assessment of Self-reported Olfactory Functioning (ASOF) test questionnaire.

Patients were categorized into SCD, naMCI, and aMCI based on clinical examination, neuropsychological test scoring, and classification guidelines using criteria presented by Jessen et al. [16] for SCD and Petersen’s criteria [17] for the diagnosis of MCI patients (aMCI and naMCI).

Based on serum TSH levels, patients were further categorized into hyperthyroidism, euthyroidism, and hypothyroidism subgroups. Participants were included if the age at the time of cognitive and olfactory testing was ≥50 years, and their blood samples for TSH were available within ±3 months of the neuropsychological assessment.

Participants were excluded if they fell into one or more of the following categories: age < 50 years, missing TSH values, presence of overt neurological disease, other types of dementia and cognitive impairment, evidence of stroke as determined by neuroradiological and clinical examinations, history of traumatic head injury, psychiatric problems causing pseudodementia, and medical conditions associated with severe cognitive impairment.

**Fig. 1:**
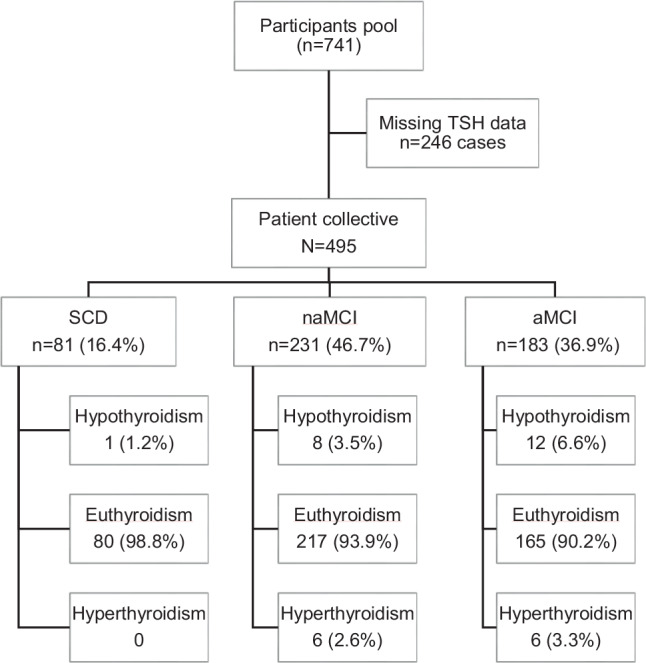
Flow chart considering the patient’s collective considering exclusion criteria and distribution of the three cognitive function groups, as well as distribution into three thyroid function states; *SCD* subjective cognitive decline; *naMCI* non-amnestic mild cognitive impairment; *aMCI* amnestic cognitive impairment; *TSH* thyroid stimulating hormone

Out of the initial 741 protocols 246 (33.2%) were excluded considering the inclusion and exclusion criteria, particularly TSH parameter completeness. This resulted in a study collective of *N* = 495 patients (81 SCD, 231 naMCI, and 183 aMCI), as shown in Fig. 1.

### Instruments

Thyroid parameters were extracted from laboratory tests performed on patients after consultation at the outpatient clinic. Serum TSH, total thyroxine (T4), free thyroxine (fT4), total triiodothyronine (T3), and free triiodothyronine (fT3) levels were determined based on standard laboratory values and reference ranges of the General Hospital of Vienna (AKH Wien), where the electrochemiluminescence immunoassay method (ECLIA) was used. The reference ranges are 0.27–4.2 µIU mL^−1^ (TSH), 0.8–1.8 ng mL^−1^ (T3), 58–124 ng mL^−1^ (T4), 2.15–4.12 pg mL^−1^ (fT3), and 0.76–1.66 ng dL^−1^ (fT4).

As TSH is the most sensitive and commonly used biomarker in determining thyroid function and as not all participants had fT3 and fT4 values available, thyroid function was assessed using only TSH levels (TSH ⇧ represents hypothyroidism, TSH ⇩ is hyperthyroidism, and TSH ⇨ is euthyroidism).

The OF was assessed using Sniffin’ Sticks© and Assessment of Self-reported Olfactory Functioning (ASOF) test for objective and subjective measurements, respectively. The Sniffin’ Sticks© test by Hummel et al. [18] is an objective nasal chemosensory performance test consisting of three subtests. This study only used the odor identification subtest and 16 common odors were presented for 3s via a felt-tip pen. The subjects were asked to identify the correct odorant from a list of four descriptors in a single choice format [18]. Scores range from 0 to 16, with ≥11 indicating intact OF and <11 reflecting hyposmia in patients over 50 years of age [19].

The ASOF test developed by Pusswald et al. [20] is a 12-item self-administered questionnaire assessing the subjective OF and its impact on quality of life. It comprises three subtests: the Subjective Olfactory Capability Scale (SOC) rating the overall olfactory ability on a scale of 0–10, the Smell-Related Problems Scale (SRP), including 5 frequency questions and the Olfactory-related Quality of Life Scale (ORQ) consisting of 6 impairment quality questions, both using a common 5‑point Likert rating scale. Cut-off scores, based on healthy control mean scores, identify decreased olfactory abilities in SOC scores ≤3, SRP scores ≤2.9, and ORQ scores ≤3.7. [20] The ASOF can be obtained from www.psimistri.com.

### Neuropsychological instruments

To evaluate the cognitive state of the participants, the following neurocognitive tests were used: the MMSE [21], VVT (version 3 0) [22] to assess visuo-constructive function, WST-IQ [23] to assess verbal intelligence and language comprehension and the NTBV, [24] a standardized instrument for the early detection of objective cognitive impairment and dementia consisting of 24 subtests, which represent the following cognitive domains: attention, executive function, language, psychomotor speed and memory. To assess the severity of depressive symptoms, the revised BDI-II [25] was used. The VVT3.0 and the NTBV can be obtained from www.psimistri.com.

### Statistical analyses

Descriptive and inferential analyses were performed using the statistical software IBM SPSS® 29.0.0 and Excel® for MacOSX. The significance level was set at alpha = 5% according to the type I error.

Considering the accumulation of type I error in multiple testing, Bonferroni adjustment α* = (α/k) (k = count of performed tests) was taken into account. The standardized effect size *r* (ranges ≥0.10 were considered as small, ≥ 0.30 as moderate, and ≥0.50 as large) according to Cohen’s classification was used to interpret the practical relevance of hypothesis testing results.

Metric parameters, at least interval-scaled ones, were characterized via mean (*M*) ± standard deviation (*SD*), minimum (min) and maximum (max). For skewed data distribution, the median (Mdn) and interquartile range (IQR, percentage range of 25–75%) were assessed. For the description of nominally scaled parameters, frequencies (*n*) and percentages (%) were presented. If appropriate, 95% confidence intervals (CI) were calculated to specify a range estimate.

In the inferential section, analyses of variances (ANOVA) were performed to test for differences in metric (at least interval-scaled) parameters between the three cognitive impairment groups. Additionally, the homogeneity of variances had to be considered using Levene’s test. In the case of heterogeneity, Welch-ANOVA was conducted. If the data were skewed, the nonparametric Kruskal-Wallis procedure was used and post hoc pairwise comparisons were conducted using the Mann-Whitney U test.

Regarding NTBV subtests, a principal component factor analysis (PCA) was performed to achieve a dimensional reduction of 24 subtests into a smaller number of independent components. This procedure was conducted utilizing an orthogonal Varimax rotation. This approach reduced redundancies and enabled weighted independent *z*-factor scores (μ = 0, σ = 1). It also allowed an adapted and standardized integration of NTBV subtests. The adequate interpretation of this dimensional reduction required two key values: the communality (h_i_^2^ ≤ 1, sum of squared factor loadings regarding one subtest considering the extracted components) and the eigenvalue (λ ≥ 1, sum of squared factor loadings regarding one extracted component considering all subtests).

## Results

### Demographics

As baseline characteristics, the data on demographics, thyroid, olfactory, and neuropsychological parameters are presented in Table 1. The proportion of female participants was 52.3% (95% CI 47.9–56.7%). The mean age of participants at testing was 69.2 ± 9.3 (min: 50.1; max: 90.4) years and no significant differences in age between cognitive subgroups (*p* = 0.195) and sex (*p* = 0.673) could be assumed. Similarly, no significant difference between cognitive subgroups regarding years of schooling was observed (*p* = 0.731).

**Table 1:** Baseline characteristics (mean, SD, median, interquartile ranges, and frequency in % and proportion) of patients according to diagnostic subgroups

Parameter		Diagnostic subgroup			Total *N* = 495
		SCD *n* = 81	naMCI *n* = 231	aMCI *n* = 183	
*Biometric/sociodemographic*					
Age (years) *Mdn*		68.6 (63.1; 76.2)	70.2 (60.8; 76.2)	71.5 (63.8; 76.8)	70.2 (62.9; 76.4)
Sex	Female	42 (51.9%)	134 (58.0%)	83 (45.4%)	259 (52.3%)
	Male	39 (48.1%)	97 (42.0%)	100 (54.6%)	236 (47.7%)
Yrs. of schooling		12 (9; 16)	12 (9; 16)	12 (8; 16)	12 (9; 16)
*Thyroid*					
TSH (µIU mL^−1^)		1.44 (1.12; 1.98)	1.62 (1.10; 2.29)	1.59 (1.06; 2.21)	1.57 (1.09; 2.22)
T3		*n* = 33	*n* = 112	*n* = 91	*n* = 236
(ng mL^−1^)		1.01 (0.92; 1.08)	1.02 (0.93; 1.12)	1.03 (0.92; 1.17)	1.02 (0.93; 1.12)
T4		*n* = 33	*n* = 112	*n* = 91	*n* = 236
(ng mL^−1^)		74.0 (65.0; 81.0)	71.0 (63.5; 84.0)	74.0 (69.0; 85.0)	73.0 (65.0; 84.0)
fT3		*n* = 28	*n* = 62	*n* = 34	*n* = 124
(pg mL^−1^)		2.89 (2.67; 3.22)	2.97 (2.76; 3.17)	2.99 (2.70; 3.28)	2.95 (2.72; 3.20)
fT4		*n* = 29	*n* = 61	*n* = 37	*n* = 127
(ng dL^−1^)		1.20 (1.11; 1.30)	1.24 (1.14; 1.33)	1.24 (1.09; 1.34)	1.23 (1.12; 1.33)
*Olfactory*					
Sniffin’ Sticks (0–16)		12.0 (11; 14)	12.0 (10; 14)	11.0 (8; 13)	12.0 (10; 13)
ASOF SOC		*n* = 80	*n* = 217	*n* = 165	*n* = 462
(0–10)		8.0 (5.0; 9.5)	8.0 (5.0; 10)	7.0 (5.0; 10)	8.0 (5.0; 10)
ASOF SRP		*n* = 81	*n* = 219	*n* = 169	*n* = 469
(1–5)		4.8 (4.0; 5)	4.8 (4.0; 5)	4.4 (3.6; 5)	4.6 (3.8; 5)
ASOF ORQ		*n* = 81	*n* = 215	*n* = 169	*n* = 465
(1–5)		5 (4.7; 5)	5 (4.7; 5)	5 (4.3; 5)	5 (4.5; 5)
*Neuropsychological*					
MMSE (0–30)		29 (28; 30)	28 (27; 29)	27 (26; 29)	28 (27; 29)
VVT 3.0		*n* = 66	*n* = 163	*n* = 130	*n* = 359
(0–10)		3.0 (1.0; 5.0)	3.0 (1.0; 6.5)	3.0 (1.0; 6.0)	3.0 (1.0; 6.0)
VVT delayed recall		*n* = 35	*n* = 95	*n* = 57	*n* = 187
(0–10)		7.0 (5.5; 9.5)	7.0 (4.5; 10)	4.0 (2.0; 7.0)	6.0 (4.0; 9.0)
WST-IQ		*n* = 80	*n* = 219	*n* = 168	*n* = 467
(μ = 100, σ = 15)		114.0 (106; 122)	110.0 (101; 118)	105.5 (99; 118)	110.0 (101; 118)
BDI-II		*n* = 80	*n* = 219	*n* = 119	*n* = 468
(0–63)		7.0 (3.5; 13.0)	10.0 (5.0; 15.0)	9.0 (5.0; 16.0)	9.0 (5.0; 15.0)
NTBV dimension		*n* = 81	*n* = 23	*n* = 183	*n* = 495
F1 Attention (−)		−0.347 (−0.734; 0.002)	0.008 (−0.556; 0.701)	−0.030 (−0.671; 0.712)	–
F2 Concentration (−)		−0.362 (−0.802; 0.063)	−0.066 (−0.612; 0.705)	−0.061 (−0.648; 0.469)	–
F3 Verbal memory (+)		0.425 (−0.146; 0.994)	0.500 (0.037; 0.909)	−0.735 (−1.304; −0.257)	–
F4 Exec. funct. skills (−)		−0.242 (−0.564; 0.253)	−0.111 (−0.644; 0.564)	−0.061 (−0.748; 0.668)	–
F5 Verbal fluency (+)		0.388 (0.065; 0.759)	−0.112 (−0.795; 0.597)	0.003 (−0.669; 0.774)	–
F6 Non-verbal fluency (−)		−0.242 (−0.515; 0.044)	−0.150 (−0.642; 0.341)	−0.265 (−0.673; 0.405)	–

*Note:* NTBV z‑scores (−) reversed, lower scores indicate better performance; (+) higher scores indicate better performance. It should be considered that the polarity of these dimensions is either negative or positive, as the NTBV subtests measure times, scores, or coefficients

*SCD* subjective cognitive decline; *naMCI* non-amnestic mild cognitive impairment; *aMCI* amnestic cognitive impairment; *Mdn* median*; Yrs* years*; TSH* thyroid-stimulating hormone;* T3* total triiodothyronine*; T4* total thyroxine*; fT3* free triiodothyronine*; fT4* free thyroxine*; ASOF* Assessment of Self-reported Olfactory Functioning*; SOC* Subjective Olfactory Capability Scale*; SRP* Smell-Related Problems Scale*; ORQ* Olfactory-related Quality of Life Scale*; MMSE* Mini Mental State Examination; *VVT* Vienna visuo-constructional test; *WST-IQ* vocabulary test (Wortschatztest-IQ); *BDI-II* Beck Depressions Inventory II; *NTBV* Neuropsychological Test Battery Vienna; *Exec funct.* executive functioning

### Thyroid parameters

**Fig. 2:**
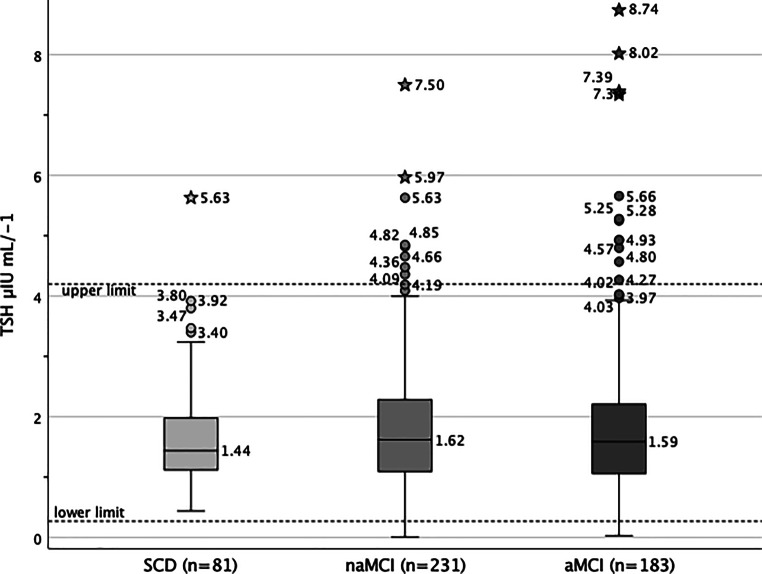
Distribution of TSH levels (*median*, outliers and extreme values) taking the reference range (dotted lines 0.27–4.20 µIU mL^−1^) into account, while considering the three cognitive subgroups

Regarding thyroid parameters, the comparison of metric laboratory values for all five hormones TSH (µIU mL^-1^), T3 (ng mL^-1^), T4 (ng mL^-1^), fT3 (pg mL^-1^), fT4 (ng dL^-1^) showed insignificant differences between the three cognitive groups, *p* ≥ 0.199. Being the most relevant parameter, TSH value distribution in the three cognitive subgroups is displayed in Fig. 2.

Furthermore, the classification of hypothyroidism, euthyroidism, and hyperthyroidism was determined based on the TSH reference ranges. The distribution of functions in the three cognitive groups is provided in Fig. 1. Accordingly, the proportion of euthyroidism was 98.8% for SCD, 93.9% for naMCI, and 90.2% for aMCI. Overall, 93.3% of all participants had normal thyroid functions.

Given the low frequency of hypothyroidism (4.3%) and hyperthyroidism (2.4%) among the participants and the absence of hyperthyroidism in the SCD group, it was considered inappropriate to perform an analysis separated into three cognitive subgroups. Therefore, OF was assessed in relation to thyroid function in the entire study cohort (*N* = 495).

### Olfactory parameters

Regarding olfaction, substantially significant differences in objective olfactory performance (Sniffin’ Sticks) were found between the three cognitive groups; *p* < 0.001; post hoc pairwise comparisons revealed that the aMCI group showed weaker olfactory performance than SCD and naMCI, *p* < 0.001, considering Bonferroni adjustment, indicating small effects, *r* = 0.23 [25]; however, concerning subjective olfactory assessment via the ASOF test (SOC, SRP, SRQ), pairwise post hoc comparisons revealed no substantial differences between cognitive subgroups. Accordingly, the extent of agreement according to rank correlation between objective (Sniffin’ Sticks as criterion) and subjective odor performances for SCD subjects was between *r*_s_ of 0.40 and 0.45 for naMCI and between *r*_s_ of 0.18 and 0.36 for aMCI.

Additionally, the proportion of hyposmic participants, taking an odor identification cut-off <11 into account, was 22.2% (13.2%; 31.3%) for SCD, 26.0% (20.3%; 31.6%) for naMCI, and 48.1% (40.8%; 55.3%) for aMCI, indicating a decreased odor performance regarding aMCI patients. In contrast, the ASOF subscales SOC, SRP, and ORQ showed no significant distributional differences comparing cognitive groups. Additionally, the proportions of subjective hyposmia ranged between 4.9% and 12.0%.

### Olfactory and thyroid functions

The consideration of OF (Sniffin’ Sticks <11 hyposmic vs. normal) and thyroid function did not reveal a significant increase in hyposmia with respect to hyperthyroidism and hypothyroidism, implying that these two entities are considered independent of each other. Similarly, the correlative relationships of Sniffin’ Sticks and ASOF subtests (SOC, SRP, ORQ) with TSH values overall (*N* = 495) and in the three diagnostic subgroups revealed only weak coincidences (*r*_s of_ −0.19 to +0.12).

Regarding the relation between the other thyroid hormones (T3, T4, fT3, and fT 4) and Sniffin’ Sticks as well as ASOF subscales, in the overall study collective (*N* = 495), only a weak negative significant relation (*p* = 0.039) was observed for fT4 (ng dL^−1^) and Sniffin’ Sticks (*r*_s_ (*n* = 127) −0.18), implying that higher olfactory performance was associated with lower fT4 levels.

### Neuropsychological functions

Neurocognitive performance was assessed via MMSE, VVT 3.0, WST-IQ, and NTBV and depression via BDI-II, as shown in Table 1. The NTBV-24 subtest results were subject to a principal component analysis (PCA) using the varimax rotation method according to Kaiser [37], achieving a dimensional reduction. The information criterion, with Kaiser–Meyer–Olkin test (KMO) = 0.838, indicates a substantial extent of information for carrying out an explorative factor analysis. These extracted components cover a total of 71.9% explained variance. The following meta-key terms can be proposed to label the six dimensions (inspired by the original NTBV domain names): (F1) perceptual functions and attention, (F2) concentration, (F3) verbal memory, (F4) executive functioning skills, (F5) verbal fluency, and (F6) nonverbal fluency (Table 2). Weighted and independent *z*-standardized factor scores were derived from these six dimensions, which are available for further analyses.

**Table 2:** Rotated component matrix, factor loadings, communality of items and eigenvalue of identified components (n = 495)

NTBV subtest	Component (dimension, factor F)						Communality
	F1	F2	F3	F4	F5	F6	h_i_^2^
Planning (maze test-NAI) time	**0.789**	0.236	−0.123	0.156	−0.067	−0.042	0.72
Planning (maze test-NAI) total/time	**−0.772**	−0.232	0.122	−0.124	0.100	0.070	0.70
TMT B	**0.696**	0.158	−0.112	0.319	−0.397	0.251	0.85
TMT B—TMT A interference	**0.627**	0.065	−0.074	0.289	−0.426	0.295	0.76
TMT A	**0.567**	0.357	−0.174	0.250	−0.119	0.001	0.56
FPT correct productions	**−0.562**	−0.230	0.221	−0.201	0.345	0.265	0.65
Digit symbol test (HAWIE-R)	**−0.504**	−0.430	0.346	−0.267	0.239	0.010	0.69
AKT time	0.446	**0.676**	−0.167	0.194	0.038	0.129	0.74
SCWT-NAI color	0.075	**0.676**	−0.115	0.262	−0.402	−0.057	0.71
Symbol counting task (C.I.)	0.235	**0.675**	−0.035	0.129	−0.232	−0.045	0.59
AKT total/time	−0.509	**−0.635**	0.203	−0.197	0.001	−0.049	0.74
Interference (C.I.) time	0.240	**0.600**	−0.118	0.436	−0.312	0.100	0.73
Interference (C.I.) total/time	−0.246	**−0.569**	0.168	−0.441	0.315	−0.053	0.71
VSRT delayed recall	−0.161	−0.049	**0.877**	−0.125	0.142	−0.060	0.84
VSRT total recall	−0.189	−0.086	**0.858**	−0.188	0.217	−0.004	0.86
VSRT immediate recall	−0.131	−0.092	**0.757**	−0.116	0.213	0.055	0.66
VSRT recognition	−0.047	−0.173	**0.727**	−0.020	−0.047	−0.144	0.58
SCWT-NAI interference	0.273	0.143	−0.148	**0.890**	−0.045	0.019	0.91
SCWT-NAI words	0.253	0.369	−0.159	**0.824**	−0.191	−0.007	0.94
SCWT-NAI total/time	−0.245	−0.349	0.159	**−0.767**	0.189	0.027	0.83
PWT total words	−0.085	−0.181	0.126	−0.224	**0.728**	0.110	0.65
SWT total words	−0.239	−0.355	0.323	−0.116	**0.570**	0.065	0.63
mBNT	−0.247	−0.144	0.154	0.003	**0.518**	−0.178	0.41
FPT perseveration	−0.006	0.031	−0.108	0.006	−0.011	**0.894**	0.81
Rotated eigenvalue (λ)	4.03	3.47	3.21	3.13	2.27	1.14	17.25
Explained variance component	16.8%	14.4%	13.4%	13.1%	9.5%	4.8%	**71.9%**

*AKT* age concentration test (lters-Konzentrations-Test); *HAWIE‑R* Hamburg Wechsler Intelligenz Test für Erwachsene Revision (which is German equivalent of WAIS‑R Wechsler Adult Intelligence Scale Revised); *TMT B* Trail Making Test Version B; *TMT A* Trail Making Test Version A; *C.I.* Cerebral Insufficiency test; *SWT* semantic verbal fluency; *mBNT* modified Boston Naming Test; *VSRT* Verbal Selective Reminding Test; *PWT* phonematic verbal fluency; *FPT* five-point-test; *NAI* Nuremberg Aging Inventory; *SCWT* Stroop Color Word Test

Bold represents factor membership

### Model for influencing factors on OF

Finally, a hierarchical modelling for influencing factors was conducted using an adjusted multiple linear regression to predict olfactory performance assessed via Sniffin’ Sticks identification subtest.

A successive increase in the explanatory value of the olfactory criteria was observed. Sniffin’ Sticks identification was analyzed through a hierarchical blockwise augmentation of the predictors and covariates so that the increase or decrease in the weight of the influencing factors can be evaluated. In the first model block, the predictor TSH (metric) was applied, in the second block the sociodemographic covariates age (metric) and sex (binary; 0 male, 1 female), in the third block neuropsychological predictor education based on years of schooling (metric discrete) and WST-IQ (metric, interval scaled), in the fourth block, depressivity (BDI-II, metric interval-scaled) and in the fifth block neurocognitive performance (NTBV-long using *z*-scored factor dimensions).

The development of coefficients by adjusted modelling for Sniffin’ Sticks olfactory identification is presented in Table 3. The tolerance value of all predictors was ≥0.61, indicating no striking multicollinearity. A normal distribution of standardized *z*-residuals and homoscedasticity could be assumed. No noticeable autocorrelations of the residuals were observed, as indicated by Durbin-Watson’s coefficient = 1.91.

**Table 3:** Regression modelling, coefficients of predictors considering olfactory identification (Sniffin’ Sticks) criterion (n = 495)

Block	Predictor	Unstand. coefficients		Stand. coeff	*t*	*p*-value	95%-CI for *B*	
		*B*	*SE*	β			LB	UB
1	(Constant)	11.219	0.258	–	43.537	<0.001	10.713	11.725
	TSH (µIU mL /‑1)	−0.020	0.118	−0.008	−0.173	0.863	−0.252	0.211
2	(Constant)	19.071	1.012	–	18.845	<0.001	17.082	21.059
	TSH (µIU mL /‑1)	0.027	0.110	0.010	0.248	0.804	−0.188	0.242
	Age (years)	−0.120	0.014	−0.357	−8.550	<0.001^**^	−0.148	−0.093
	Sex	0.741	0.263	0.118	2.820	0.005^**^	0.225	1.257
3	(Constant)	16.902	1.543	–	10.956	<0.001	13.871	19.933
	TSH µIU mL /‑1	0.042	0.110	0.016	0.382	0.703	−0.174	0.258
	Age (years)	−0.123	0.014	−0.364	−8.462	<0.001^**^	−0.151	−0.094
	Sex	0.796	0.265	0.127	3.008	0.003^**^	0.276	1.316
	Years of schooling	−0.023	0.039	−0.030	−0.582	0.561	−0.099	0.054
	WST-IQ	0.023	0.012	0.094	1.876	0.061	−0.001	0.048
4	(Constant)	16.803	1.582	–	10.622	<0.001	13.695	19.911
	TSH (µIU mL /‑1)	0.040	0.110	0.015	0.366	0.714	−0.176	0.257
	Age (years)	−0.122	0.015	−0.363	−8.426	<0.001^**^	−0.151	−0.094
	Sex	0.787	0.267	0.125	2.949	0.003^**^	0.263	1.311
	Years of schooling	−0.022	0.039	−0.029	−0.562	0.574	−0.099	0.055
	WST-IQ	0.024	0.013	0.095	1.891	0.059	−0.001	0.048
	BDI-II	0.005	0.016	0.012	0.289	0.773	−0.027	0.037
5	(Constant)	14.335	1.578	–	9.087	<0.001	11.236	27.435
	TSH (µIU mL /‑1)	0.054	0.105	0.021	0.515	0.607	−0.152	0.260
	Age (years)	−0.069	0.017	−0.204	−4.126	<0.001^**^	−0.102	−0.036
	Sex	0.216	0.280	0.034	0.773	0.440	−0.334	0.766
	Years of schooling	−0.049	0.038	−0.063	−1.292	0.197	−0.123	0.025
	WST-IQ	0.017	0.013	0.070	1.377	0.169	−0.007	0.042
	BDI-II	0.010	0.016	0.025	0.601	0.548	−0.022	0.041
	F1 Attention (−)	−0.250	0.142	−0.080	−1.762	0.079	−0.528	0.029
	F2 Concentration (−)	−0.373	0.130	−0.119	−2.866	0.004^**^	−0.628	−0.117
	F3 Verbal memory (+)	1.026	0.147	0.327	6.988	<0.001^**^	0.738	1.315
	F4 Exec. funct. skills (−)	−0.003	0.128	−0.001	−0.025	0.980	−0.254	0.248
	F5 Verbal fluency (+)	−0.014	0.135	−0.004	−0.101	0.929	−0.279	0.252
	F6 Nonverbal fluency (−)	−0.203	0.127	−0.065	−1.604	0.109	−0.452	0.046

Note: (−) reversed, lower scores indicate better performance; (+) higher scores indicate better performance; *R*^2^ = 24.4%, *R*^2^_adj._ = 22.5%; *F *(12, 482) = 12.952

*TSH* thyroid-stimulating hormone*; WST-IQ* Wortschatztest-IQ; *BDI-II* Beck-Depressions-Inventory II; *Exec funct.* executive functioning; *SE* standard error; *CI* confidence interval

*p* < 0.001, ^**^p ≤ 0.01

The global model summary revealed an explanatory value of *R*^2^_adj._ = 22.5%, *p* < 0.001, indicating a significant contribution of predictors for olfactory identification (Sniffin’ Sticks). Increasing age, as seen in the last fifth model block, with a small weight (β = −0.204, *p* < 0.001) appears as a risk factor for decreased olfactory identification, whereas a better performance of the NTBV dimensions verbal memory (F3) with a moderate weight (β = +0.327, *p* < 0.001), concentration (F2) with a small weight (β = +0.119, *p* = 0.004) provide increased smell identification. All other independent variables and covariates were not significantly contributing to predicting objective olfactory performance. In particular, TSH did not reveal a substantial weight.

## Discussion

Thyroid hormones, OF, and cognitive impairment may be subject to a relationship that is not yet fully understood. This study, however, did not reveal a substantial relation between cognitive subgroups and thyroid function.

There was significant difference in OF in the three cognitive subgroups where hyposmic participants with aMCI were more affected (48.1%) than the naMCI (26.0%) and SCD (22.2%) patients. This finding is consistent with previous study results where olfactory identification scores using Sniffin’ Sticks were progressively lower starting from cognitively normal aged, followed by SCD, MCI, and AD patients [26]. Tahmasebi et al. said that odor identification was significantly lower in aMCI patients than in naMCI patients [27].

Regarding the relation between thyroid hormones and olfaction, no association between objective olfactory identification and thyroid function assessed via TSH was observed. Furthermore, in this blockwise hierarchical regression model, TSH plays no substantial role in influencing OF. It can be concluded that the thyroid function has no relation to olfactory impairment.

Concerning the remaining thyroid hormones (T3, T4, fT3, fT4), no significant coincidence to objective and subjective olfactory assessments, apart from fT4, could be stated. A weak negatively significant relationship *r*_s_ (−0.18) was observed between Sniffin’ Sticks performance and fT4, implying that higher fT4 levels are associated with lower Sniffin’ Sticks scores. This result is not in line with a previous study conducted by Günbey et al., where olfactory parameters had no significant relationship with fT4 but were significantly associated with fT3 [14]; however, there have been associations with higher fT4 levels and increased risk of dementia [10, 11, 28, 29]. This finding leaves room for debate on whether fT4 may be a mutual biomarker for OD and cognitive impairment but it may also be that this result is a statistical artifact and thus irrelevant.

Furthermore, regarding subjective olfactory assessment using the ASOF test, in the SCD subgroup, the agreement between objective and subjective assessments was comparatively the highest, suggesting that this group can assess itself more appropriately. Thus, this finding matches those of previous studies, where lack of olfactory self-awareness in MCI could serve as a diagnostic marker for early AD [27, 30].

Increasing age is associated with decreasing olfactory performance (β = −0.204). This result is consistent with previous studies with larger sample sizes. Sorokowska et al. showed that participants over the age of 70 years had lower olfactory identification scores than participants between 20 and 60 years old [31]. Oleszkiewicz further validated these findings in a study with a sample size of over 9000 participants where there was a marked decline in olfactory performance between the ages of 60 and 71 years [32]. Interestingly, our study did not reveal the participants’ sex to be an influencing factor. Although studies with larger sample sizes have shown that females have an olfactory advantage, the effect size was very small and the difference in performance was very subtle [32, 33].

Concerning neurocognitive abilities, a better performance of the NTBV dimensions verbal memory (F3) with a moderate weight (β = +0.327) and concentration (F2) with a small weight (β = +0.119) provide increased smell identification ability. Previous studies in a patient population with early cognitive decline showed that among different cognitive subdomains, dysfunction in olfactory identification showed the strongest correlation with memory. Within various memory subdomains, this dysfunction showed the highest relation with verbal memory, specifically immediate verbal recall [26, 34]. Our results support previous findings, indicating that OD is more likely linked to verbal memory loss than other cognitive domains. This point suggests that olfactory impairment could facilitate the early detection of memory decline [34].

Furthermore, our study showed, albeit with a small weight, that a decrease in concentration and attention negatively affects olfactory performance. A study by Bergmann et al. found similar results, where associations between OF and concentration, attention, and verbal memory were observed in cognitively impaired individuals [35]. These findings imply that the combination of olfactory identification testing and neuropsychological test batteries might be used in the detection of early AD.

Our study faced several limitations, including insufficient cases with varied thyroid functions in each cognitive subgroup and zero hyperthyroid subjects among SCD. Only four cases had data for all five thyroid parameters due to the changes in laboratory guidelines from the late 1990s to the mid-2000s. Incomplete and missing data, coupled with variations in thyroid testing protocols, may have impacted statistical power, introducing bias. Additionally, TSH variations could be masked by unaccounted thyroid medications. Olfactory performance results might be influenced by factors like smoking or interventions. Further research with larger samples and comprehensive thyroid hormone variations is crucial to exploring the thyroid-olfactory-cognitive relationship accurately.

Furthermore, in terms of comorbidity our study ignored potential comorbidities typical in a routine care dataset, especially in a population with an average age of 70 years, including metabolic (e.g., diabetes, vitamin B12), toxic (e.g., smoking, medication), genetic (e.g., Apolipo-Proteine-E (APOE) ε4), and other states (e.g., trauma, respiratory tract infection, COVID, otolaryngological and psychiatric disorders), or vascular health with impact on olfactory functions. Including these comorbidities might change the results. In addition, the observed associations may only be relevant for subjects attending a memory clinic due to memory problems (i.e., SCD, MCI and potential future AD patients), not for other neurological disorders or normal cognitive aging at the community level. We did not explore dementia risk of olfaction or thyroid dysfunction as a cross-sectional design was used and patients were not followed-up.

In conclusion, among the thyroid hormones, only fT4 showed a weak significant effect on OF, with higher fT4 levels correlating with lower identification scores. The TSH levels showed no relationship with OF, which requires further research for confirmation. When comparing olfactory self-reports and objective functions, the SCD group showed the highest agreement, while naMCI and aMCI showed no significant associations. In addition, increasing age and decreasing performance in the NTBV verbal memory and concentration dimensions emerged as significant predictors of OD. These findings suggest that neurocognitive test performance may serve as an early predictor of OD in the spectrum of cognitive impairment.
